# Low Doses of Ionizing Radiation Promote Tumor Growth and Metastasis by Enhancing Angiogenesis

**DOI:** 10.1371/journal.pone.0011222

**Published:** 2010-06-21

**Authors:** Inês Sofia Vala, Leila R. Martins, Natsuko Imaizumi, Raquel J. Nunes, José Rino, François Kuonen, Lara M. Carvalho, Curzio Rüegg, Isabel Monteiro Grillo, João Taborda Barata, Marc Mareel, Susana Constantino Rosa Santos

**Affiliations:** 1 Angiogenesis Unit, Instituto de Medicina Molecular, Faculdade de Medicina da Universidade de Lisboa, Lisbon, Portugal; 2 Cancer Biology Unit, Instituto de Medicina Molecular, Faculdade de Medicina da Universidade de Lisboa, Lisbon, Portugal; 3 Division of Experimental Oncology, Centre Pluridisciplinaire d'Oncologie, Faculty of Biology and Medicine, University of Lausanne, and NCCR Molecular Oncology ISREC-EPFL, Epalinges, Switzerland; 4 Bioimaging Unit, Instituto de Medicina Molecular, Faculdade de Medicina da Universidade de Lisboa, Lisbon, Portugal; 5 Zebrafish Unit, Instituto de Medicina Molecular, Faculdade de Medicina da Universidade de Lisboa, Lisbon, Portugal; 6 Serviço de Radioterapia do Hospital de Santa Maria, Lisbon, Portugal and Instituto de Medicina Molecular, Faculdade de Medicina da Universidade de Lisboa, Lisbon, Portugal; 7 Department of Radiotherapy, Ghent University Hospital, Ghent, Belgium; UIC, United States of America

## Abstract

Radiotherapy is a widely used treatment option in cancer. However, recent evidence suggests that doses of ionizing radiation (IR) delivered inside the tumor target volume, during fractionated radiotherapy, can promote tumor invasion and metastasis. Furthermore, the tissues that surround the tumor area are also exposed to low doses of IR that are lower than those delivered inside the tumor mass, because external radiotherapy is delivered to the tumor through multiple radiation beams, in order to prevent damage of organs at risk. The biological effects of these low doses of IR on the healthy tissue surrounding the tumor area, and in particular on the vasculature remain largely to be determined. We found that doses of IR lower or equal to 0.8 Gy enhance endothelial cell migration without impinging on cell proliferation or survival. Moreover, we show that low-dose IR induces a rapid phosphorylation of several endothelial cell proteins, including the Vascular Endothelial Growth Factor (VEGF) Receptor-2 and induces VEGF production in hypoxia mimicking conditions. By activating the VEGF Receptor-2, low-dose IR enhances endothelial cell migration and prevents endothelial cell death promoted by an anti-angiogenic drug, bevacizumab. In addition, we observed that low-dose IR accelerates embryonic angiogenic sprouting during zebrafish development and promotes adult angiogenesis during zebrafish fin regeneration and in the murine Matrigel assay. Using murine experimental models of leukemia and orthotopic breast cancer, we show that low-dose IR promotes tumor growth and metastasis and that these effects were prevented by the administration of a VEGF receptor-tyrosine kinase inhibitor immediately before IR exposure. These findings demonstrate a new mechanism to the understanding of the potential pro-metastatic effect of IR and may provide a new rationale basis to the improvement of current radiotherapy protocols.

## Introduction

Radiotherapy is a widely used local treatment for malignant tumors, characterized by uncontrolled growth and the ability of invading adjacent tissues and metastasize. While radiotherapy has been classically viewed to exert its therapeutic effect by killing tumor cells, emerging evidence indicates that effects extend beyond cancer cell death. Ionizing radiation (IR) changes the microenvironment, contributing to anti-tumor effects of radiotherapy [1]. However, there are clinical and experimental observations indicating that IR might promote a metastatic behavior of cancer cells and that the irradiated host microenvironment might exert tumor-promoting effects [1], [2]. Therefore, a careful analysis of the putative tumor-promoting and pro-metastatic effect of IR is imperative, as radiotherapy is an essential part of cancer treatment. Several tumor-associated host cells including endothelial cells, leukocytes, macrophages, fibroblasts, myofibroblasts and nerve cells populate the tumor microenvironment. Recently, different studies have focused on the mechanisms by which IR activates cellular targets potentially contributing to invasion and metastasis [3], [4], [5], [6]. Doses of IR causing such stimulating effects are classically delivered inside the tumor target volume in daily small fractions in order to limit damage of healthy tissues and until a potentially curative dose has accumulated inside the tumor volume. Furthermore, the delivery in small fractions and the isodose distributions of external beam radiotherapy result in lower doses of IR outside the tumor target volume. The biological effects of these low doses of IR on the healthy tissue surrounding the tumor area, and in particular on the vasculature remain largely to be determined.

Here we report that, *in vitro*, IR doses lower than 0.8 Gy do not cause cell cycle arrest or apoptosis. We found that low-dose IR led to the phosphorylation of several cellular proteins including VEGF receptor-2 (VEGFR-2). By activating VEGFR-2, low-dose IR enhances endothelial cell migration and prevents endothelial cell death promoted by the VEGF-neutralizing antibody bevacizumab. Also, under hypoxic conditions low-dose IR upregulates VEGF. We further show that, in zebrafish, low-dose IR accelerates sprouting angiogenesis during development and enhances the angiogenic response during caudal fin regeneration. Using different mouse models, we show that low-dose IR promotes angiogenesis resulting in accelerated tumor growth and metastasis formation in a VEGFR-dependent manner.

These observations provide novel insights into the mechanisms involved in the pro-metastatic effect of IR and open novel therapeutic perspectives for the improvement of current radiotherapy protocols.

## Results

### Low-dose IR promotes endothelial cell migration without causing cell cycle arrest or apoptosis

**Figure 1:**
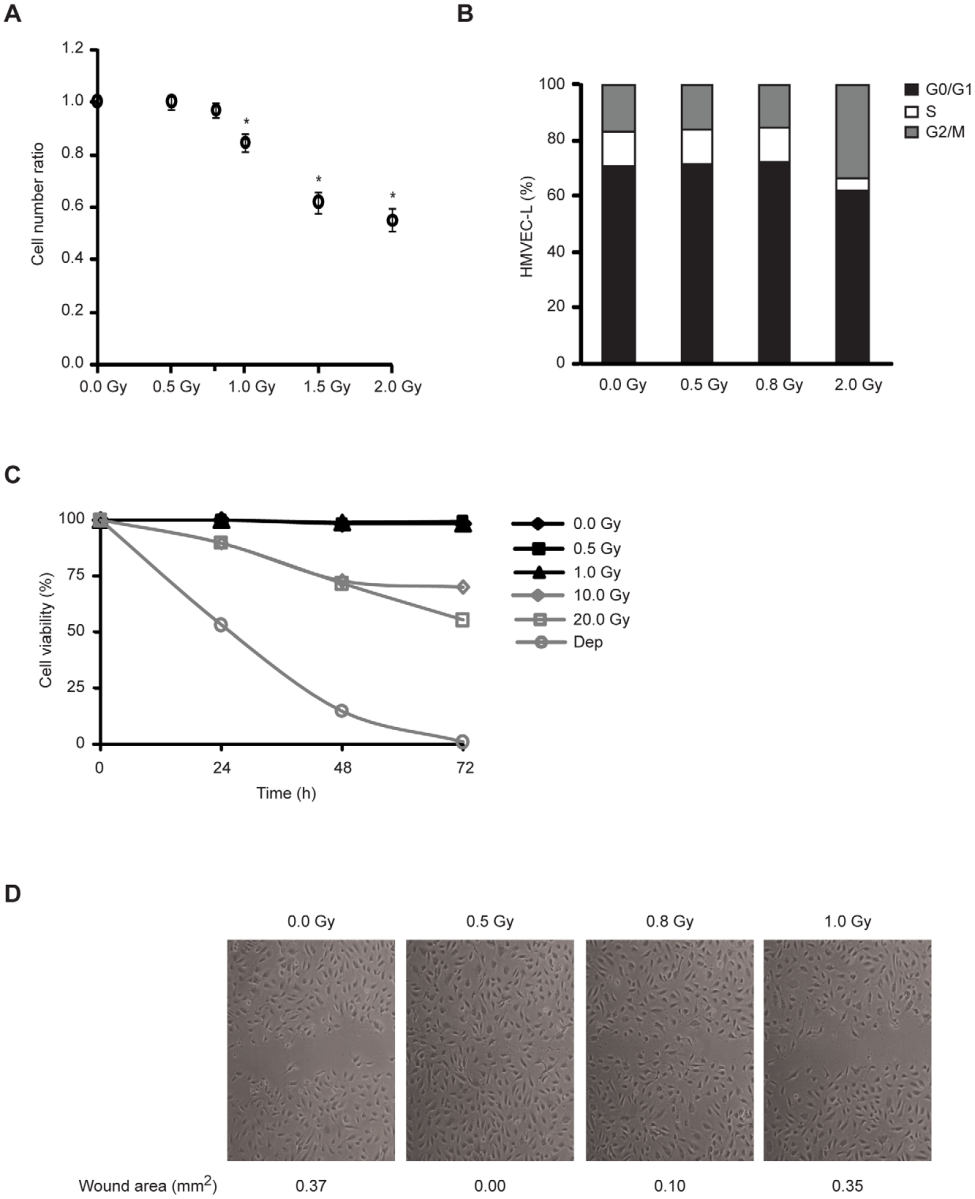
Low-dose IR promotes endothelial cell migration without causing cell cycle arrest or apoptosis. (A) HMVEC-L were plated at equal densities and, after 12 h, left untreated or exposed to 0.5, 0.8, 1.0, 1.5 and 2.0 Gy. After 72 h, the cells were counted using a Nucleocounter. The values (means ± s.d.) represent the ratio between cell number of irradiated and non-irradiated conditions and are derived from four independent experiments. ^*^ *P*<0.02. (B) HMVEC-L were exposed or not to 0.5, 0.8 and 2.0 Gy. Cell cycle profiles were assessed by flow cytometric analysis after 72 h of culture. Data are representative of three independent experiments. (C) The percentage of apoptotic cells was determined by flow cytometry at the indicated time. Cells cultured without serum (Dep) were used as cell death control. Values are given as the percentage of viable cells (Annexin V, PI negative) remaining in culture. Data are shown as mean in triplicate culture and are representative of three independent experiments. (D) Confluent monolayers of HMVEC-L were subjected to *in vitro* wound healing and exposed or not to 0.5, 0.8 or 1.0 Gy. Photographs were taken immediately (not shown) and 9 h after wounding. Quantification of the wound area (in mm^2^) is presented below the images. Data are representative of five independent experiments.

To investigate the biological effects of low doses of IR on endothelial cells, we exposed lung human microvascular endothelial cells (HMVEC-L) to doses lower than 2.0 Gy and assess effects on cell proliferation, survival and migration. We found that 0.5 and 0.8 Gy did not modulate HMVEC-L proliferation, while doses higher than 1.0 Gy caused a significant decrease of their proliferation rate (Figure 1A). Accordingly, detailed analysis of cell cycle profile did not reveal any significant changes in phase distribution in cells irradiated with 0.5 or 0.8 Gy (Figure 1B). The impact of low-dose IR on HMVEC-L survival was also evaluated. No effect on cell viability was detected at doses up to 1.0 Gy, whereas higher doses (i.e. 10.0 and 20.0 Gy) caused increased cell death (Figure 1C). Moreover, we assessed the migration of HMVEC-L exposed to low-dose IR by the scratch wound healing assay. Doses of 0.5 and 0.8 Gy stimulated HMVEC-L migration and wound closure, whereas cells irradiated with 1.0 Gy presented a migration rate similar to non-irradiated controls (Figure 1D). To examine whether low-dose IR nevertheless induced DNA double-strand breaks we monitored H2AX phosphorylation [7]. As early as 30 min after irradiation the γ-H2AX foci were detected in a dose dependent manner in the nuclei of irradiated cells (Figure S1). At 12 h post-irradiation, γ-H2AX was no longer detectable in cells irradiated at low doses reflecting effective DNA double-strand break repair.

Taken together these results indicate that doses of 0.5 or 0.8 Gy promote endothelial cell migration and, in spite of induction of DNA double-strand breaks they do not impinge on endothelial cell proliferation or survival.

### Low-dose IR activates the PI3K/Akt and MEK/ERK signaling pathways and prevents apoptosis induced by PI3K or MEK inhibition

**Figure 2:**
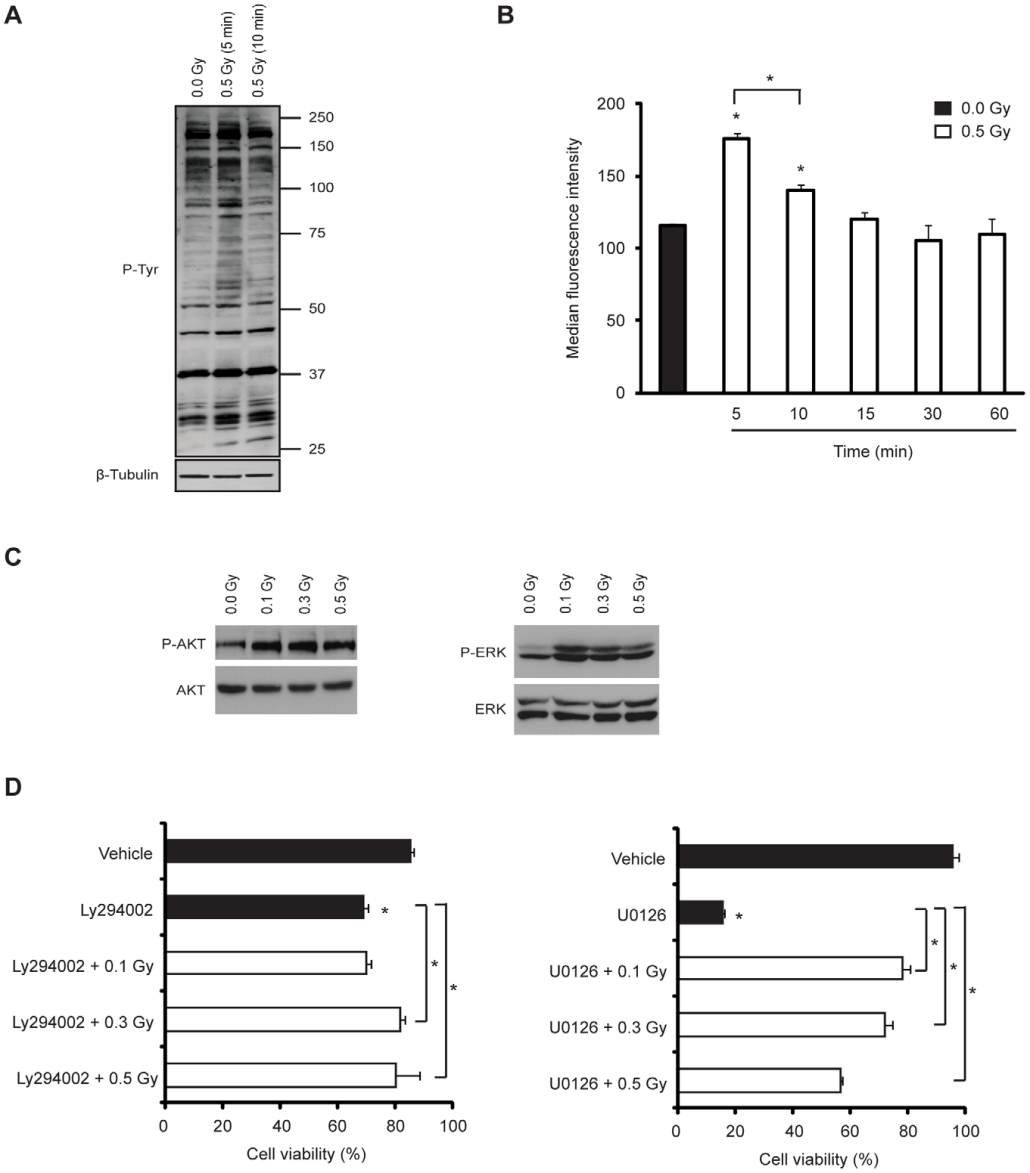
Low-dose IR activates PI3K/Akt and MEK/ERK pathways and prevents apoptosis induced by their inhibition. (A and B) HMVEC-L were exposed or not to 0.5 Gy and incubated for the time indicated. (A) Representative blots from three independent experiments. Top, Tyrosine phosphorylation Western blot; Bottom, β-tubulin Western blot to verify equal sample loading. (B) Tyrosine phosphorylation levels were assessed by flow cytometry. The values (means ± s.d.) represent the ratio between median fluorescence intensity of irradiated cells and non-irradiated cells and are derived from four independent experiments. (C) Representative blots from three independent experiments. Western blot analysis of total-, phospho-Akt (P-AKT) (left) and total-, phospho-ERK (P-ERK) (right) of HMVEC-L exposed or not to 0.1, 0.3 or 0.5 Gy. The levels of Akt and ERK phosphorylation were assessed after 5 or 60 min post-irradiation, respectively. (D) HMVEC-L were incubated in the presence or absence of a specific inhibitor of PI3K (Ly294002-50 µM) (left) or MAPK (U0126-10 µM) (right) and then exposed or not to 0.1, 0.3 or 0.5 Gy. Cells cultured with vehicle alone were used as a control. Cells were double stained with Annexin-V and propidium iodide at 36 h post-irradiation. The percentage of apoptotic cells was assessed by flow cytometry. The values (means ± s.d.) are given as the percentage of viable cells and are derived from four independent experiments. ^*^ *P*<0.03.

Next, we investigated whether low-dose IR could activate intracellular signaling pathways associated with cell survival and migration. To this end, HMVEC-L were exposed to 0.5 Gy and global protein phosphorylation was analysed. A transient increase in tyrosine phosphorylation of multiple proteins was evident 5 min post-irradiation, but no longer detected after 10 min (Figure 2A and 2B), suggesting the rapid triggering of tyrosine kinase activity upon irradiation. Next, we evaluated the activation of PI3K/Akt and MEK/ERK signaling pathways. We observed a slight but significant increase in phosphorylation of Akt and ERK upon exposure to 0.1, 0.3 or 0.5 Gy (Figure 2C). To assess the functional relevance of IR-induced activation of Akt and ERK, HMVEC-L cultured in the presence of specific inhibitors of PI3K (Ly294002) or MEK (U0126) were exposed or not to 0.1, 0.3 or 0.5 Gy. Treatment with either of these inhibitors decreased HMVEC-L viability, although to different extents. Remarkably, low-dose IR treatment significantly protected HMVEC-L from death induced by the signaling inhibitors (Figure 2 D).

### Low-dose IR promotes endothelial cell migration and protects microvasculature from bevacizumab-induced cell death by activating VEGFR-2

**Figure 3:**
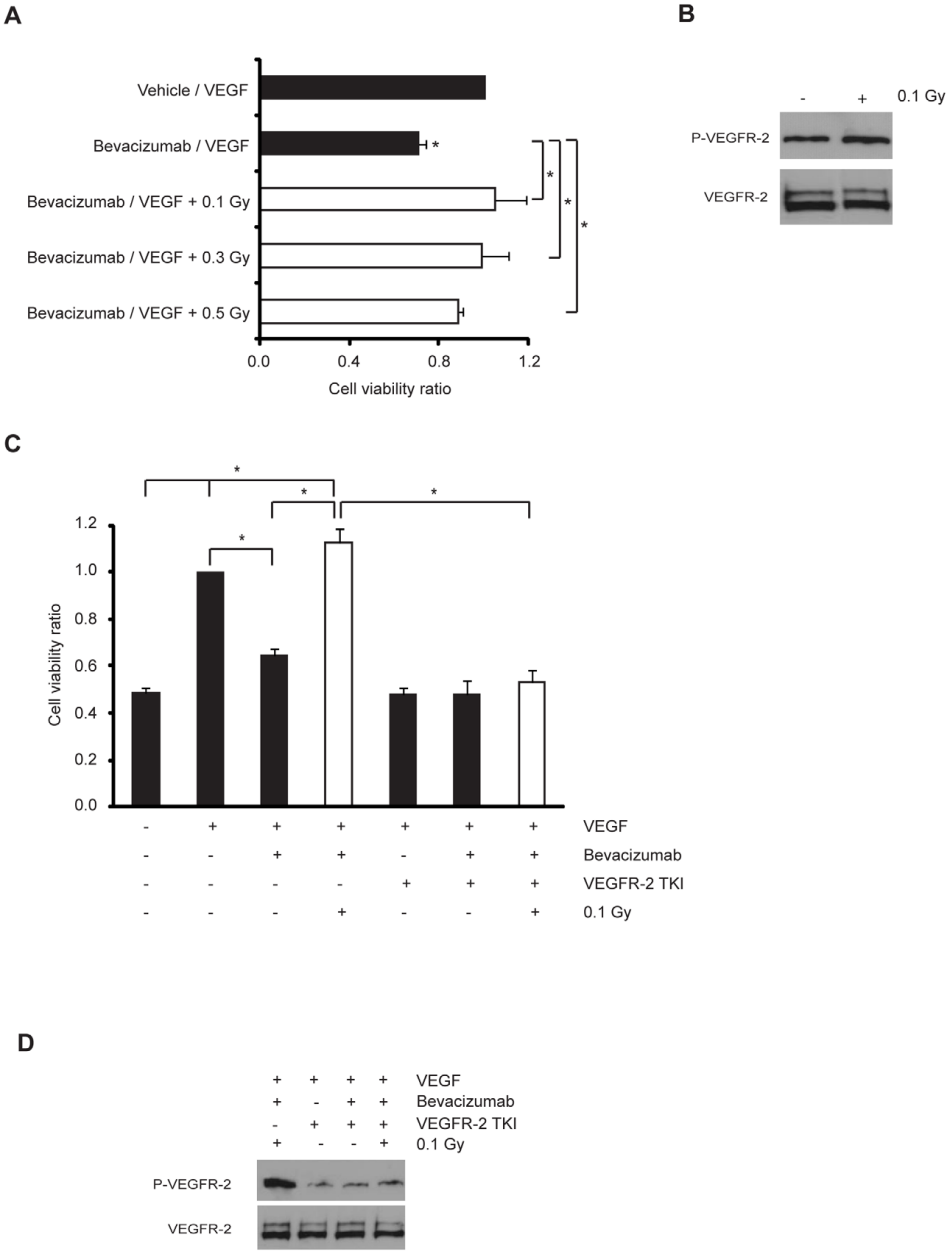
Low-dose IR protects microvasculature from bevacizumab-induced cell death by inducing VEGFR-2 activation. (A) Cells were cultured without serum for 12 h and incubated with vehicle/VEGF (20 ng/ml) or bevacizumab (0.25 mg/ml)/VEGF (20 ng/ml) mixtures. Then, cells were exposed or not to 0.1, 0.3 or 0.5 Gy and the percentage of apoptotic cells was assessed by flow cytometry at 48 h post-irradiation. Data (means ± s.d.) represent the ratio between cell viability percentage of each experimental condition and control condition and are derived from four independent experiments. (B) Representative blots from four independent experiments. Cells were exposed or not to 0.1 Gy. Western blot analysis of total- and phospho-VEGFR-2. (C) Cells were cultured without serum for 12 h and treated or not with VEGFR-2 tyrosine kinase inhibitor (TKI at 300 nM) for 2 h and stimulated or not with VEGF (20 ng/ml) or bevacizumab (0.25 mg/ml)/VEGF (20 ng/ml) mixture. Then, cells were exposed or not to 0.1 Gy. The percentage of apoptotic cells was assessed by flow cytometry at 48 h post-irradiation. Data (means ± s.d.) represent the ratio between cell viability percentage of each experimental condition and control condition and are derived from four independent experiments. ^*^ *P*<0.03. (D) Representative blots from four independent experiments. Western-blot analysis of total- and phospho-VEGFR-2 (P-VEGFR-2) of HMVEC-L cultured without serum for 12 h and treated or not with VEGFR-2 tyrosine kinase inhibitor (TKI at 300 nM) for 2 h and stimulated with VEGF (20 ng/ml) or bevacizumab (0.25 mg/ml)/VEGF (20 ng/ml) mixture. (B and D) The level of VEGFR-2 phosphorylation was assessed after 15 min post-irradiation.

The previous results incited us to test the effect of low-dose IR on HMVEC-L death induced by the inhibition of VEGF, a main survival factor for endothelial cells and a target for anti-angiogenic therapies. HMVEC-L were incubated with bevacizumab (Avastin®), a monoclonal antibody that inhibits the biologic activity of VEGF, before exposure to low-dose IR. Bevacizumab treatment reduced HMVEC-L viability and low-dose IR significantly protected against bevacizumab-induced death (Figure 3A). The same low-dose IR did not protect against cell death induced by different chemotherapeutic agents (Figure S2). Based on its ability of low-dose IR to induce protein phosphorylation, we hypothesized that low-dose IR counteracted the pro-apoptotic effect of bevacizumab by activating VEGF receptor-2 (VEGFR-2). Consistent with this hypothesis, we found a significant increase in VEGFR-2 phosphorylation in response to 0.1 Gy (Figure 3B). To test the functional impact of VEGFR-2 activation, HMVEC-L were cultured in the presence of bevacizumab and treated or not with a VEGFR-2 tyrosine kinase inhibitor (TKI) prior to 0.1 Gy of IR. We found that the protective effect was completely abrogated by treatment with VEGFR-2 TKI (Figure 3C). Molecularly, we demonstrated that the VEGFR-2 TKI, in contrast to bevacizumab, inhibited VEGFR-2 phosphorylation induced by 0.1 Gy. (Figure 3D). Since VEGFR-2 activation has been reported to promote endothelial cell migration [8], we investigated whether low-dose IR promotes endothelial cell migration (Figure 1D) through the activation of VEGFR-2. HMVEC-L were treated or not with VEGFR-2 TKI prior to 0.5 Gy of IR and cell migration was assessed by the scratch wound healing assay. We found that the pro-migratory effect promoted by low dose IR was significantly abrogated by treatment with VEGFR-2 TKI (Figure S3).

Taken together these results demonstrate that low-dose IR enhances endothelial migration and prevents endothelial cell death promoted by the VEGF-neutralizing antibody bevacizumab, by inducing VEGFR-2 phosphorylation.

### Low-dose IR induces VEGF expression in endothelial cells under conditions mimicking hypoxia

**Figure 4:**
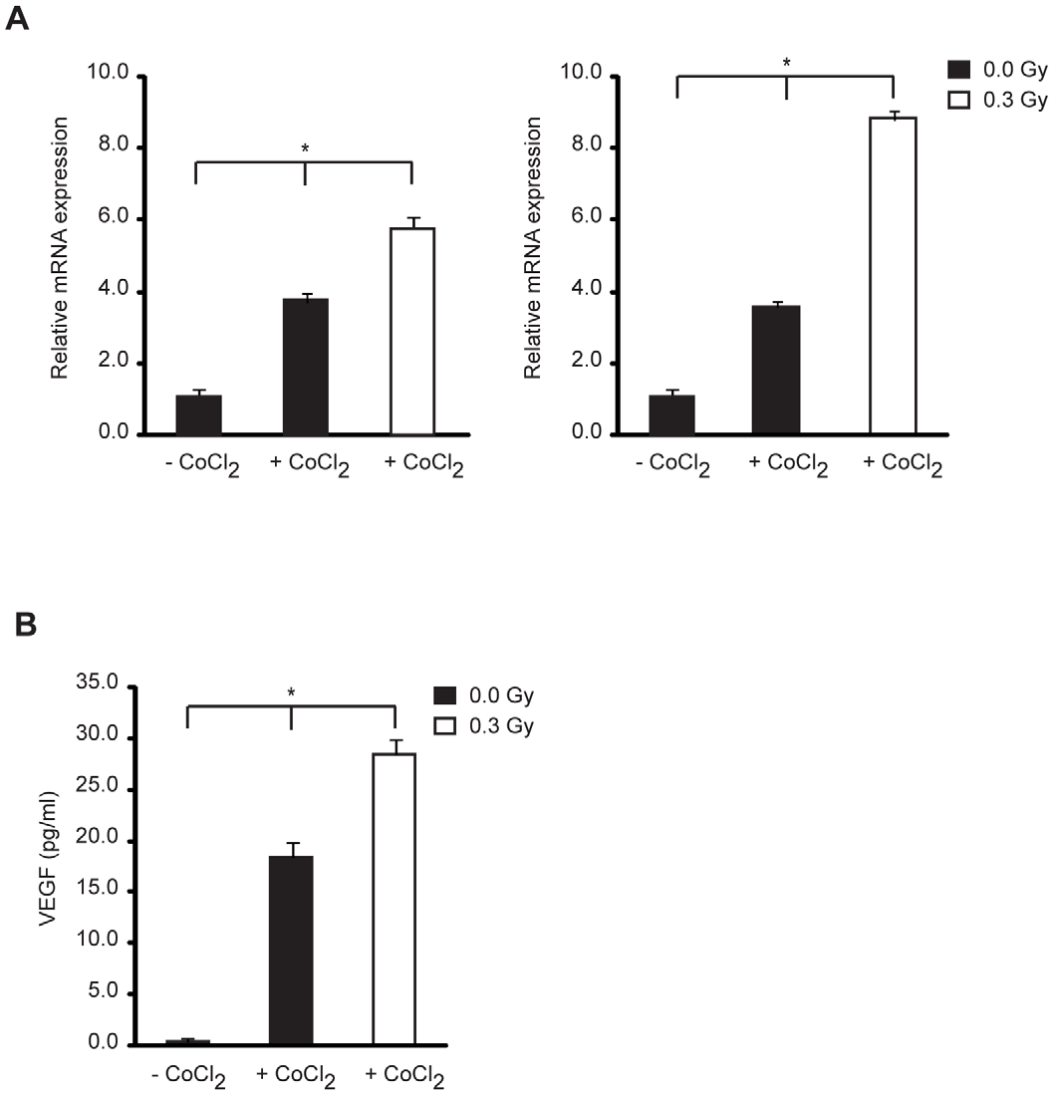
Low dose IR enhances hypoxia-induced VEGF expression. Cells were cultured with or without CoCl_2_ (150 µM) in normoxia to mimic hypoxic conditions and immediately exposed or not to 0.3 Gy of IR. (A) 4 h (left graph) and 12 h (right graph) post-irradiation, *VEGF* mRNA was quantified by qRT-PCR. Data (means ± s.d.) represent the fold change in gene expression relative to the internal calibrator (−CoCl2) in triplicate measurements and are representative of three independent experiments. (B) 72 h post-irradiation, VEGF protein was assessed by VEGF ELISA Kit. Data (means ± s.d.) indicate the VEGF concentration in quadruplicate measurements and are representative of three independent experiments. ^*^ *P*<0.03.

We next asked the question whether low-dose IR modulates the expression of VEGF in endothelial cells. Under normoxic conditions, endothelial cells constitutively express very low levels of VEGF [9]. Low-dose IR did not induce VEGF expression under normoxic conditions (data not shown). However, in HMVEC-L cultures first exposed to cobalt chloride (CoCl_2_), which mimics hypoxic conditions, and subsequently treated with 0.3 Gy of IR, we observed a greater increase of VEGF mRNA levels compared to cells treated with CoCl_2_ alone (Figure 4A). Consistent with increase VEGF mRNA expression, we also observed increased levels of secreted VEGF protein (Figure 4B).

These data indicate that low-dose IR enhances VEGF expression in endothelial cells under hypoxia-mimicking conditions.

### Low-dose IR accelerates angiogenic sprouting during zebrafish embryonic development and enhances angiogenesis during zebrafish fin regeneration

**Figure 5:**
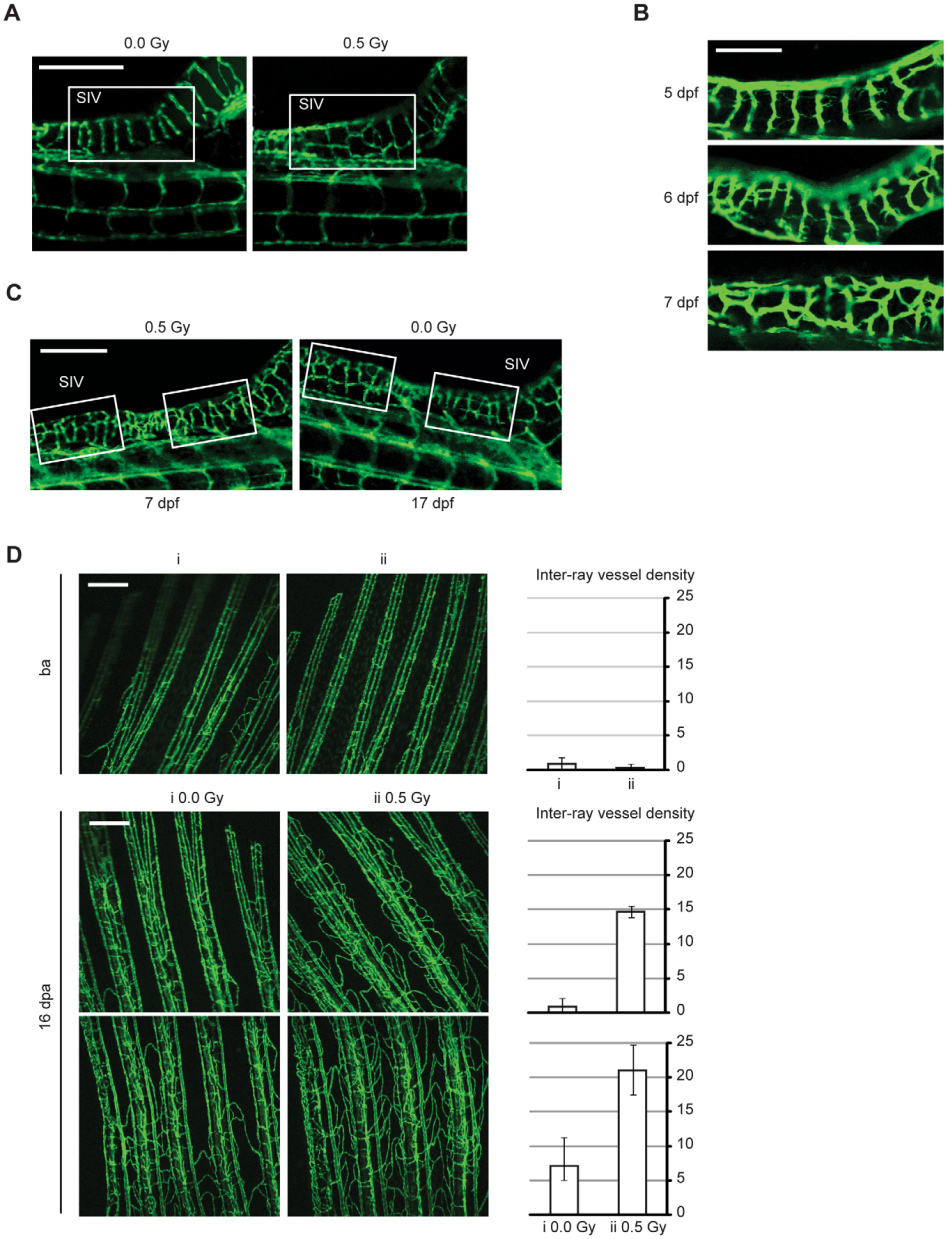
Low-dose IR accelerates angiogenic sprouting during zebrafish embryonic development and enhances angiogenesis during fin regeneration. (A–C) Live zebrafish embryos were exposed or not to 0.5 Gy IR 3 d post-fertilization (dpf). Representative images of Sub-Intestinal Vessels (SIV), from (A) a non-irradiated and irradiated zebrafish at 7 dpf; (B) an irradiated zebrafish at 5 (top), 6 (middle) and 7 dpf (bottom); (C) an irradiated zebrafish at 7 dpf and a non-irradiated zebrafish at 17 dpf. Scale bars, 250 µm (A and C), 100 µm (B). (D) Fli1:EGFP adult zebrafish caudal fins were amputated at mid-fin level, exposed or not to 0.5 Gy of IR and then allowed to recover. Representative images from vasculature of two zebrafish fins (i and ii) before amputation ensure they had identical vasculature (top). Representative images from vasculature of two different fin areas of the same zebrafish, 16 days post-amputation (dpa), with or without low-dose IR treatment (middle and bottom). Each image was quantified for inter-ray vessel density. Data are shown as mean and error bars indicate maximum and minimum values. Images are representative of 10 zebrafish in five independent experiments. Scale bars, 250 µm.

To ascertain whether low-dose IR was able to enhance angiogenesis *in vivo*, we used the transgenic zebrafish fli1∶EGFP, which allow intra vital imaging of the vasculature through fli1 gene promoter-driven expression of GFP in endothelial cells. The first blood vessels formed in zebrafish embryo arise through vasculogenesis. The smaller vessels are then formed through angiogenic sprouting from these large vessels [10]. Fli1∶EGFP zebrafish embryos were exposed to 0.5 Gy IR 3 days post-fertilization (dpf), a time of angiogenic sprouting, and the formed vasculature was examined 7 dpf. All non-irradiated embryos (n = 70) showed vertical, parallel-oriented and regularly spaced Sub-Intestinal Vessels (SIV), while 52 out of 70 irradiated embryos (74%), showed more and irregularly-shaped vessels between larger SIV (Figure 5A). We also found that these numerous vessels were formed by sprouting angiogenesis (Figure 5B). At a later time point (17 dpf), SIV vascular pattern was identical in non-irradiated embryos (Figure 5C), indicating that low-dose IR accelerated angiogenic sprouting without causing excessive vessel formation.

To test whether low-dose IR also stimulated angiogenesis in adult animals, adult fli1∶EGFP zebrafish were subjected to caudal fin amputation at mid-fin level, exposed or not to 0.5 Gy of IR and then allowed to recover. Zebrafish caudal fin growth is accompanied by active angiogenesis [11]. Results showed a striking increase in the inter-ray vessel density in the irradiated regenerating fin (Figure 5D).

Taken together, these data demonstrate that low-dose IR accelerates sprouting angiogenesis from SIV during zebrafish embryonic development, and enhances the angiogenic response during fin regeneration.

### Low-dose IR promotes angiogenesis in the murine Matrigel plug assay

**Figure 6:**
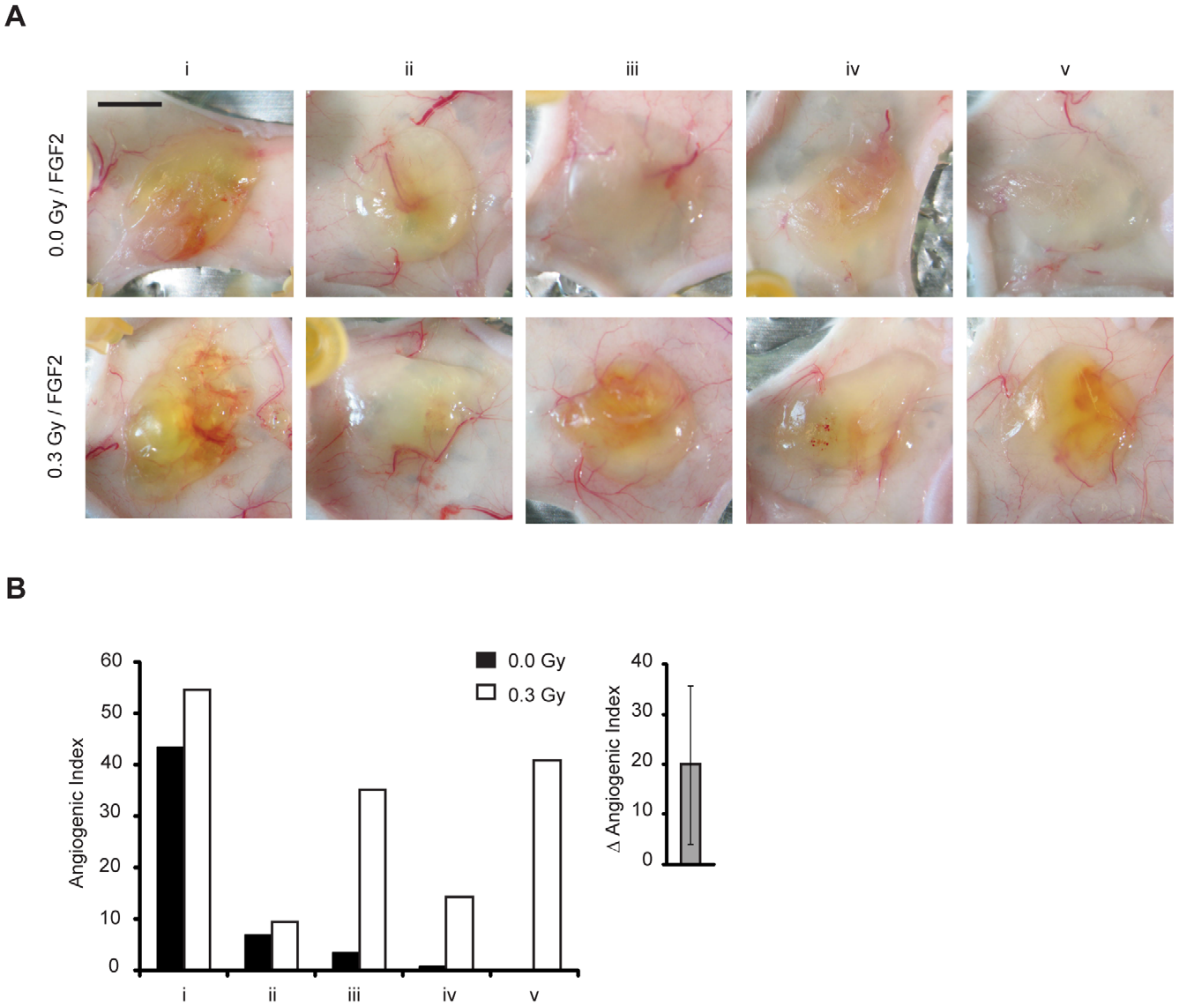
Low-dose IR enhances angiogenesis in Matrigel plug assay. Angiogenesis was induced by injection of growth factor-depleted Matrigel with FGF2. (A) Macroscopic evaluation of the Matrigel plugs explanted 5 d after injection in non-irradiated and 0.3 Gy preirradiated area. Scale bars, 500 µm. (B) Angiogenesis was quantified by determining the angiogenic index. Inset shows the mean difference of angiogenic index between paired mice. * *P*<0.025.

To test whether low-dose IR also stimulates angiogenesis in mammals, we used the murine Matrigel plug angiogenesis assay [12]. Athymic Swiss *nu/nu* mice were locally irradiated (lower-right back side) with 0.3 Gy, and growth-factor-depleted Matrigel plugs supplemented with fibroblast growth factor 2 (FGF2) were implanted 24 h later, within irradiated or non-irradiated tissue (the contralateral non-irradiated side was used as matched controls). Matrigel plugs were analyzed 5 days later, a time point where angiogenesis is naturally heterogeneous and not fully formed, thereby facilitating the detection of stimulatory effects, while at later times (7 to 10 d) angiogenesis is more homogenous and robust, which may mask stimulatory effects. Consistently, the degree of angiogenesis ranged from high to low across different plugs. Tissue pre-irradiation enhanced angiogenesis, albeit to different extents in the individual mice (Figure 6A and 6B).

These data demonstrate that low-dose IR significantly promotes angiogenesis in adult mice.

### Low-dose IR promotes acceleration of tumor growth and metastasis in a VEGF receptor-dependent manner

**Figure 7:**
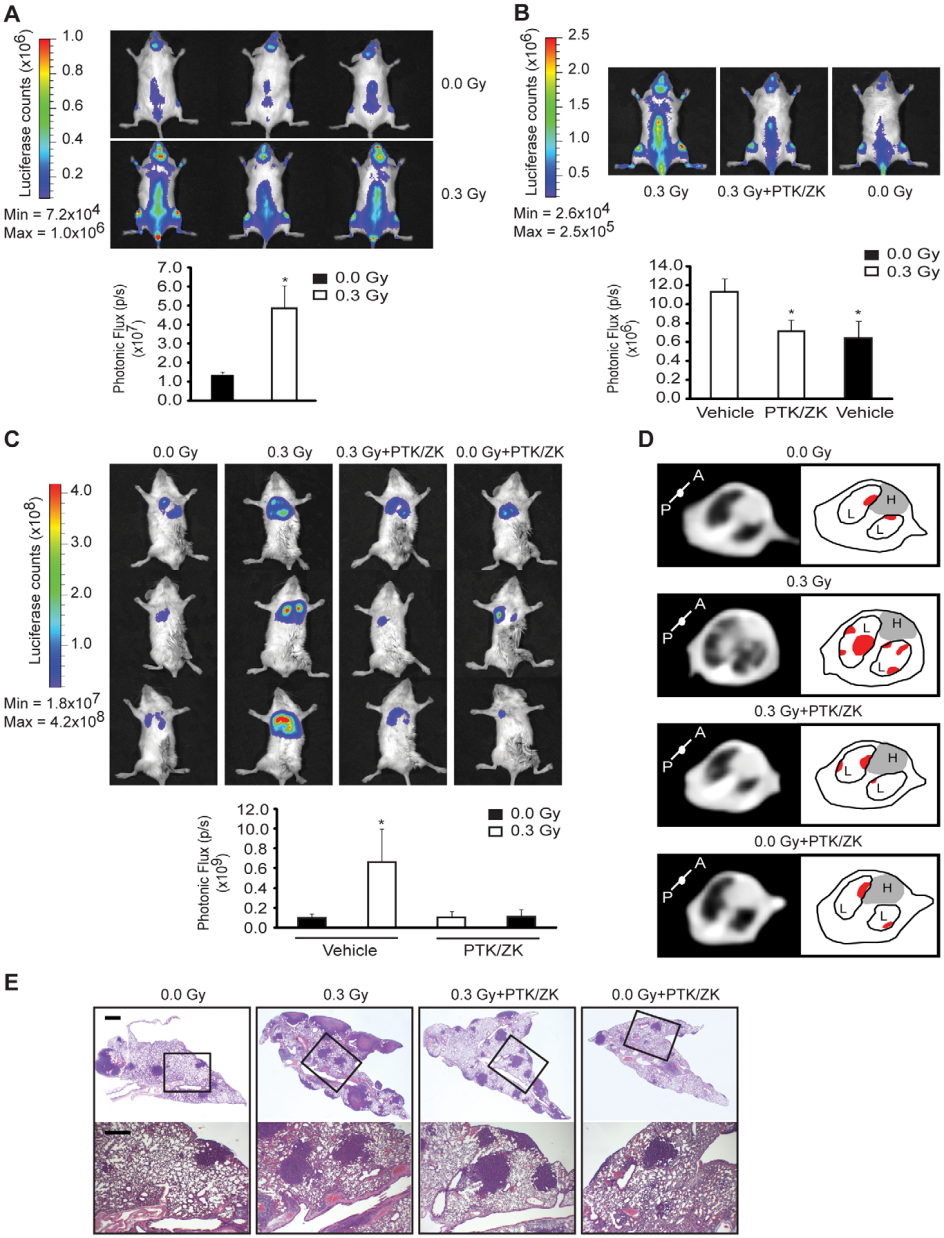
Low-dose IR promotes acceleration of tumor growth and metastasis in a VEGF receptor-dependent manner. (A) NOD-SCID mice were irradiated or not with 0.3 Gy and 22 h later injected with MOLT-4 cells (B) NOD-SCID mice were pre-treated with PTK/ZK (100 mg/Kg) or PTK/ZK vehicle, 2 h later irradiated or not with 0.3 Gy and after 22 h injected intravenously with MOLT-4 cells. (A and B) 14 d post-injection, the tumor burden was quantified by bioimaging. n = 3 mice per group. The values (means ± s.d.) are representative of three independent experiments. Dorsal images from representative mice are shown. ^*^ *P*<0.02. (C) NOD-SCID IL-2R gamma^null^ mice were treated with PTK/ZK (100 mg/Kg) or PTK/ZK vehicle, 2 h later irradiated or not with 0.3 Gy and after 22 h injected with 4T1 cells into the mammary fat pad. 20 d post-injection, the primary tumor was removed and lung metastases were quantified by bioimaging. n = 3 mice per group. The values (means ± s.d.) are representative of three independent experiments. ^*^ *P*<0.05. Ventral images from representative mice were shown. (D) Representative images of CT cross section of lung area, from one mouse per experimental condition, showing pulmonary nodules in both lobes of lungs (left). Schematic illustration (right) of the pulmonary nodules (in red) located in both lobes (L) of the lungs. The gray area represents the heart (H). (E) Representative lung sections from one mouse per experimental condition stained with H&E. Scale bars, 1 mm (top), 0.5 mm (bottom).

We asked whether low-dose IR had an impact in promoting tumor growth and dissemination. Six weeks-old NOD-SCID mice were irradiated or not with 0.3 Gy and subsequently injected intravenously with MOLT-4 cells. After 14 d, irradiated mice showed a significant increase in MOLT-4 tumor burden when compared to non-irradiated animals (Figure 7A).

Next, we investigated whether VEGFR activation could be involved in the acceleration of tumor growth promoted by low-dose IR. To this purpose, we administered the VEGF receptor tyrosine kinase inhibitor, PTK/ZK, 2h before irradiation with 0.3 Gy, and MOLT-4 cells were injected 24 h later. At this time point PTK/ZK is no longer active *in vivo*, thereby excluding possible direct effects on leukemia cells. After 14 d, leukemia burden in irradiated mice treated with PTK/ZK was significantly lower than in irradiated animals treated with vehicle only, and was similar to the tumor burden observed in non-irradiated mice (Figure 7B).

We also investigated whether low-dose IR could also promote tumor metastasis by using the 4T1 orthotopic implantation mouse model of spontaneous breast cancer metastasis to the lung [13]. NOD-SCID IL-2Rgamma^null^ mice were treated or not with PTK/ZK and 2 h later exposed or not to 0.3 Gy, whole body IR. After 22 h, 5×10^4^ 4T1 cells were injected into the 4^th^ mammary fatpad of the mice in all four groups. Non-irradiated mice treated with PTK/ZK were also used as a control, to ensure that the single PTK/ZK treatment administered 24 h before 4T1 cell injection would not affect tumor growth. Twenty days post-transplantation, no difference in primary tumor growth between the groups of mice was found (data not shown). Primary tumors were surgically removed and a significantly higher bioluminescent signal was detected in the chest region of the irradiated group when compared to other groups, where the signal was similar (Figure 7C). By Computed Tomography (CT) scan, we found more nodules in both lobes of irradiated lungs, when compared to other groups (Figure 7D). To confirm the bioluminescent and CT results, lungs were analysed by histology at necropsy. Accordingly, number of metastasized 4T1 cells in the lungs of experimental mice groups was similar, except in the irradiated one, in which we observed an increased number and size of lung metastases (Figure 7E).

Taken together, these results indicate that low-dose IR promotes acceleration of tumor growth and enhances the metastatic spreading in a VEGFR-dependent manner.

## Discussion

There is growing evidence in the literature indicating that IR may have pro-metastatic effects [2]. It is generally assumed that tumor progression towards metastasis, during or after therapy, is due to the appearance of resistant tumor cells. However, several studies indicate that radiotherapy also rapidly and persistently alters the tumor microenvironment. IR induces the production of pro-angiogenic molecules [4], which may activate the microenvironment, including the vasculature. In accordance, anti-angiogenic approaches enhance anti-tumor effects of IR [14], [15], [16], [17]. On the other hand, while adjuvant radiotherapy significantly improves local tumor control, recurrences within a preirradiated field are associated with higher risk of local invasion and metastasis and with poor prognosis [18], [19], [20].

Furthermore, the tissues that surround the tumor area are also exposed to low doses of IR that are lower than those delivered inside the tumor mass, because external radiotherapy is delivered to the tumor through multiple radiation beams, in order to prevent damage of organs at risk. Our present data suggest that these low doses of IR promote angiogenesis and consequently tumor burden and metastasis. We found that doses of 0.8 Gy or lower do not cause cell cycle arrest or apoptosis. Molecularly, they led to rapid phosphorylation of several cellular proteins including Akt and ERK, which play relevant roles in microvascular endothelial cell function. Interestingly, our results suggest that there is not a specific low IR-dose that is most effective in activating specific cellular proteins and molecular response induced by low dose-IR, since effects are dependent not only on the IR dose but also on the molecular target. Doses of 0.1 and 0.3 Gy induce a similar phosphorylation of AKT that is greater than the phosphorylation induced by 0.5 Gy. On the other hand 0.1 Gy is more effective in inducing phosphorylation of ERK, compared to 0.3 and 0.5 Gy doses. Moreover, low-dose IR can protect endothelial cells against apoptosis induced by inhibiting these signaling pathways. Thus, low-dose IR might disrupt the balance between survival and apoptosis by activating pro-survival signaling proteins, thereby favoring angiogenesis. These findings do not contradict the concept that the pro-apoptotic effects of radiation on endothelial cells contribute to antitumoral treatment, since these effects occur only at high IR doses [21]. Rather, they suggest that at given doses and at time points during radiotherapy, IR might enhance the formation of new vessels, thereby favoring metastatic spreading.

We also demonstrated a protective effect of low-dose IR against endothelial cell death induced by bevacizumab, an anti-VEGF monoclonal antibody, currently used in the clinic as anti-angiogenic drug in combination with chemotherapy [22]. VEGF inhibition by bevacizumab can prevent binding to its receptors, in particular VEGFR-2 which is the main pro-angiogenic VEGF receptor on endothelial cells [22], [23]. Consistently, we found that low-dose IR activates VEGFR-2 and the protective effect of IR against bevacizumab-mediated VEGF inhibition was completely abrogated by treatment with a VEGFR-2 TKI. Therefore, we propose that low-dose IR might prevent endothelial cells death promoted by bevacizumab through a mechanism involving VEGFR-2 phosphorylation. We observed that low-dose IR enhanced endothelial cell migration by activating VEGFR-2. While this result is partially consistent with previous reports demonstrating that VEGFR-2 activation promotes endothelial cell migration [8], it also suggests that low-dose IR might modulate other molecules involved in endothelial cell migration since the treatment with a VEGFR-2 TKI does not completely suppress IR-induced migration.

Our data also showed that low-dose IR induces VEGF transcription and consequent protein expression in endothelial cells under hypoxia-mimicking conditions. CoCl_2_ induces the expression of the transcription factor hypoxia-inducible factor 1 (HIF-1) α subunit, which leads to the increased transcription of genes involved in the initiation and progression of angiogenesis [9]. In normoxia, HIF1 activity is low and consequently the endothelial cells express basal levels of VEGF [9]. We found that these levels were not changed by low-dose IR. However, after CoCl_2_ treatment and low-dose IR exposure, the expression of VEGF is higher than in non-irradiated cells treated with CoCl_2_ alone. Although our data do not explain the mechanism by which low-dose IR upregulates VEGF under hypoxic conditions, this finding is of potential clinical relevance since hypoxic areas are naturally present both within tumor and in its periphery [24]. The exposure of hypoxic areas in the healthy tissues surrounding the tumor to low doses of IR may further enhance VEGF expression and favor tumor cell escape.

Taken together, our *in vitro* results suggest that low-dose IR promote endothelial cell events, including the activation of VEGFR, critical to the angiogenic process. Accordingly, we found that low-dose IR increases vessel density in adult fli1∶EGFP zebrafish and mice and accelerates vessel formation by inducing angiogenic sprouting in fli1∶EGFP embryos. This pro-angiogenic effect of low dose IR may be of clinical relevance, since low doses of IR promoted tumor spreading of leukaemic cells and lung metastasis formation of breast cancer in mice. According to our *in vitro* results where we demonstrated an activation of VEGFR-2 by low-dose IR, the VEGF receptor tyrosine kinase inhibitor PTK/ZK, was administered before irradiation in order to avoid the effect of low dose IR in inducing the phosphorylation of VEGFR in mice irradiated with 0.3 Gy. It is described that the activation of VEGFR leads to a rapid activation of different cellular proteins and consequently to the *de novo* mRNA and protein expression of mediators involved in the angiogenic response. This is strongly supported by the data we obtained in an *in vitro* microarray study, where several transcripts encoding for proteins required for angiogenesis are induced upon low-dose IR delivery (data not shown). However, since tumor cells also express VEGFR, PTK/ZK would directly interfere with their viability thereby confounding the results of the experiment. For that reason, the MOLT-4 or 4T1 cells were injected 24 h after PTK/ZK treatment, a time point this inhibitor is no longer active *in vivo*. We observed that in both mice models the experimental group treated with PTK/ZK presented tumor spreading and lung metastasis formation similar to the unirradiated experimental group, consistent with the hypothesis that IR promoted tumor spreading of leukaemic cells and lung metastasis through a mechanism involving the activation of VEGFR. Two putative vascular beds might be involved in this effect: the vascular beds that surrounds the primary tumor (which provides an efficient exit route for tumor cells); and the vasculature located in the metastatic organ (which will favor initial tumor cells homing and supply oxygen and nutrients necessary for the proliferation and survival of metastatic tumor cells).

In conclusion, our findings provide new insights into tumor radiobiology, identifying the pro-angiogenic effects that low-dose IR might have on the vasculature surrounding the tumoral mass, and thus adding a clinically relevant element to the interpretation of dosimetry in radiotherapy.

## Materials and Methods

### Ethics Statement

All animals experiments were carried out in accordance with protocols approved by the Animal Care Committee of the IMM (permit numbers AEC_004, AEC_005 and AEC_007).

### Cell culture and reagents

Lung Human Microvascular Endothelial Cells (HMEC-L) were purchased from Cambrex and cultured according to manufacturer's instructions. Cells were used up to passage 6. T-cell leukemia-derived MOLT-4 cells and 4T1 breast carcinoma cells (American Type Culture Collection), both stably expressing luciferase, were cultured in RPMI and 10% FBS. Ly294002 (50 µM), VEGF (20 ng/ml) and CoCl_2_ (150 µM) were purchased from Sigma. U0126 (10 µM) was purchased from New England Biolabs. VEGF receptor-2 tyrosine kinase inhibitor (VEGFR-2 TKI) (70nM) was purchased from Calbiochem. PTK787/ZK222584 (PTK/ZK) (100 mg kg^−1^) was provided by Novartis Pharma AG, Basel, Switzerland. Bevacizumab (0.25 mg/ml), 5-FU (5 µg/ml), gemcitabine (0.08 µM) and paclitaxel (3 nM) were provided by the Oncology Service of Santa Maria Hospital, Lisbon, Portugal.

### Irradiation

HMVEC-L cultures, anesthetized mice or zebrafish were transferred to an acrylic phantom box in order to achieve a certain thickness. A computed tomography (CT) scan (Somatom Sensation, Siemens) was performed and a volumetric acquisition was carried out; acquired images were reconstructed with axial slices width of 1 mm, and cross sectional data was transferred to the image processing system work station for contouring the planning target volume (PTV). The radiotherapy plan was devised on a dedicated 3D planning system (PLATO, Nucletron) using an isocentric dose distribution of two opposite fields (0°, 180°) at 6 MV energy, normalized to a reference point. IR delivery was performed at room temperature using a linear accelerator x-rays photon beam (Varian Clinac 2100 CD) operating at a dose rate of 300 MU/min. A 0.6 cm^3^ PTW farmer ionizing chamber, connected to UNIDOS electrometers, was used to validate the IR doses calculated by PLATO, according to the IAEA TRS-398 protocol. We obtained, in average, differences lower than 2% between the experimental and the PLATO planning system dose values.

### Wound healing assay

HMVEC-L were plated to confluence and wounds created in the monolayer by scraping the plate with a pipette tip. Monolayers were irradiated or not and photographed immediately after wounding and 9 h later.

### Proliferation and cell cycle analysis

Cells were counted using a nucleocounter (ChemoMetec) according to manufacturer's instructions. Cell cycle assays were performed as described [25] and analyzed using a FACS Calibur (Beckton Dickinson) and ModFit LT 2.0 Software.

### Apoptosis analysis

HMEC-L were plated at equal densities, after 12 h incubated in the different experimental conditions and stained with Annexin V-FITC (Boehringer Manheim) and propidium iodide (PI) (Interchim). The percentage of apoptotic cells (Annexin V positive, PI negative and positive) was determined by flow cytometry (FACS Calibur, Beckton Dickinson) and Flowjo 6.4.7 Software. Results are shown as the percentage of viable cells (Annexin V, PI negative).

### Immunofluorescence analysis

HMEC-L were cultured on gelatin-coated glass coverslips. The cells were fixed, permeabilized, and incubated with antibody to γ-H2AX (from Upstate Biotechnology Inc.) followed by incubation with Alexa Fluor 488 (Molecular Probes). The samples were counterstained with DAPI and analyzed by fluorescence microscopy (Axioplan Microscope, Zeiss).

### Flow cytometry analysis

The cells were fixed, permeabilized and stained with the antibody to phospho-tyrosine (Santa Cruz Biotechnology) followed by incubation with Alexa Fluor 488 or 594 (Molecular Probes). We acquired cells using FACS Calibur (Beckton Dickinson) and analyzed data using Flowjo 6.4.7 Software.

### Western-blot analysis

Whole protein extracts were prepared as described [26]. Blots were incubated with antibodies to phospho-tyrosine, phospho-ERK (Tyr204), ERK, phospho Akt (Ser473), Akt (Santa Cruz Biotechnology), VEGFR-2 (Calbiochem) or β-tubulin (Sigma).

### Quantitative RT-PCR

For quantitative RT-PCR, total RNA was isolated using the QiaShredder and RNeasy (Qiagen) system. For each sample, 1 µg RNA was reverse transcribed into cDNA (Superscript II Kit, Invitrogen). *VEGF* mRNA levels were measured by RT-PCR (TAQMAN) on the ABI Prism® 7900HT Sequence Detection System (Applied Biosystems) using specific primers and probes (forward primer: 5′-CCAGCACATAGGAGAGATGAGCTT-3′, reverse primer: 5′-CGCCTCGGCTTGTCACA-3′, probe: 6-FAM-5′-ACAGCACAACAAATGTGAATGCAGACCAAA-3′-TAMRA). The housekeeping gene used to normalize the samples in TAQMAN assay was the 18S (Human 18S rRNA-20×, Applied Biosystems). Real time PCR program consisted of an initial denaturation step at 95°C for 10 min followed by 40 cycles at 95°C for 15 s and at 60°C for 1 min. The relative quantification of VEGF mRNA in HMEC-L was performed according to the comparative method (2 −DDCt; Applied Biosystems User Bulletin no. 2P/N 4303859), with −CoCl2 condition as internal calibrator. The formula used is 2 −DDCt  = 2 −[DCt(sample)−DCt(calibrator)], where DCt(sample) = Ct(sample)−Ct(reference gene). For the internal calibrator, DDCt = 0 and 2 0 = 1. For the remaining samples, the value of 2 −DDCt indicates the fold change in gene expression relative to the calibrator. DCt value for each sample is the average of triplicates.

### ELISA assay

Conditioned medium from the different experimental conditions was stored at −80°C and VEGF concentrations were measured following manufacturer's instructions using the human VEGF-ELISA kit (Calbiochem).

### Zebrafish

The transgenic zebrafish fli1∶EGFP were obtained from the Zebrafish International Resource Center and maintained under standard conditions [27] with institutional animal care. Before irradiation or imaging, adults and embryos zebrafish were anesthetized using 0.61 mM and 0.31 mM of tricaine, respectively. Caudal fins were acquired on a Zeiss LSM 5 Live microscope using a 10×/0.30 objective and a solid state 488 nm laser in conjunction with a LP 505 nm filter. Zebrafish embryos images were acquired on a Zeiss LSM 510 META using a 20×/0.80 objective and the 488 nm laser line of an Ar laser in conjunction with a LP 505 nm filter. The vessel density was calculated with Image J (NIH) using threshold segmentation and histogram analysis to measure blood vessel areas per total area of selected regions of interest (inter-ray regions).

### Mice and mouse procedures

Athymic Swiss *nu/nu*, NOD-SCID and NOD-SCID IL-2R gamma^null^ mice were purchased from the Harlan Laboratories (Madison, WI, USA), the pathogen-free facility of the Instituto de Medicina Molecular (IMM) and the Jackson Laboratory (Bar Harbor, Maine, USA), respectively. Matrigel plug angiogenesis assay was adapted from the original method [12]. Briefly, 24 h after 0.3 Gy local radiation of 8 week-old Athymic Swiss *nu/nu* female mice (lower-right back side), two Matrigel plugs (400 µl/plug) supplemented with FGF-2 (500 ng/ml, PeproTech EC Ltd) and Heparin (3 U/ml, Sigma) were implanted in the irradiated dorsal region of the mice or in the contralateral non-irradiated side (control plug) of same mouse. Five days after Matrigel implantation, the plugs were removed and photographed using 0.56× magnification. Angiogenesis was reported as the angiogenic index (mean number of red pixels per the total number of pixels in the area of interest) using the Image J (NIH). 6-week-old NOD-SCID mice were injected intravenously (i.v.) with 20×10^6^ MOLT-4 cells. 6 week-old NOD-SCID IL-2R gamma^null^ female mice were injected with 5×10^4^ 4T1 cells suspended in 50% PBS/50% Matrigel (from BD Biosciences) into the 4^th^ mammary fat pad. PTK/ZK or its vehicle (polyethylene glycol-300 from Sigma) was administered by intragastric gavage. For imaging studies, mice were anesthetized and injected with d-luciferin at 150 mg kg^−1^ (Xenogen) intraperitoneally (i.p). We imaged photonic emission with the *In Vivo* Imaging System (IVIS, Xenogen) with a collection time of 180 s for MOLT-4 experiments or 10 s for 4T1 experiment. We quantified tumor bioluminescence by integrating the photonic flux (photons per second) through a region encircling each tumor, as determined by the LIVING IMAGES Software package (Xenogen). We evaluated lung metastasis by Computed Tomography (CT) scan. Acquired images were reconstructed with axial slices width of 1 mm. For histological analysis, the mice lungs were dissected, immersion-fixed overnight, at room temperature, in 4% paraformaldehyde and then embedded in paraffin. Paraffin-embedded sections with 3 µm thickness were counterstained with hematoxylin and eosin (H&E) using traditional methods.

### Statistical analysis

Numeric data were analyzed for statistical significance using Mann-Whitney test for comparison of means with GraphPad Prism 5 Software. Two-tailed unpaired and paired Student's *t*-test was also used for tumor and Matrigel plug assay experiments, respectively. P<0.05 was considered significant.

## Supporting Information

Figure S1 Low doses of IR induce phosphorylation of H2AX. Cells were exposed or not to 0.3, 0.5, 1.0 and 2.0 Gy and γ-H2AX foci, marking DNA damage, were visualized, by immunofluorescence microscopy, after 30 min and 12 h post-irradiation. γ-H2AX foci are shown in green and nuclei are stained with DAPI (in blue). Magnification, 400×.
(1.07 MB TIF)

Figure S2 Low doses of IR do not protect the microvasculature from 5-FU-, gemcitabine- or paclitaxel-induced cell death. HMVEC-L were cultured for 12 h and treated or not with (A) 5-FU (5 µ g/ml); (B) gemcitabine (0.08 µ M); (C) paclitaxel (3 nM) and then exposed or not to 0.1, 0.3 or 0.5 Gy. Non-irradiated cells cultured with vehicle alone were used as a control. Cells were double stained with Annexin-V and propidium iodide at 48 h post-irradiation. The percentage of apoptotic cells was assessed by flow cytometry. Data (means ± s.d.) represent the ratio between the cell viability percentage of each experimental condition and the control condition and are derived from four independent experiments.
(0.38 MB TIF)

Figure S3 Low-dose IR promotes endothelial cell migration by activating VEGFR-2. Confluent monolayers of HMVEC-L were treated or not with VEGFR-2 tyrosine kinase inhibitor (TKI at 300 nM) for 2 h, subjected to in vitro wound healing and next exposed or not to 0.5 Gy. The quantification of the wound area (in mm2) was assessed 9h after wounding. Data (means ± s.d.) indicate the percentage of wound recovery in quadruplicate measurements and are representative of three independent experiments. *P<0.05.
(0.13 MB TIF)
