# Supplementary figures and images for: Low Doses of Ionizing Radiation Promote Tumor Growth and Metastasis by Enhancing Angiogenesis
Source: PLoS One. 2010 Jun 21;5(6):e11222. doi: 10.1371/journal.pone.0011222 (PMC2888592; doi:10.1371/journal.pone.0011222)

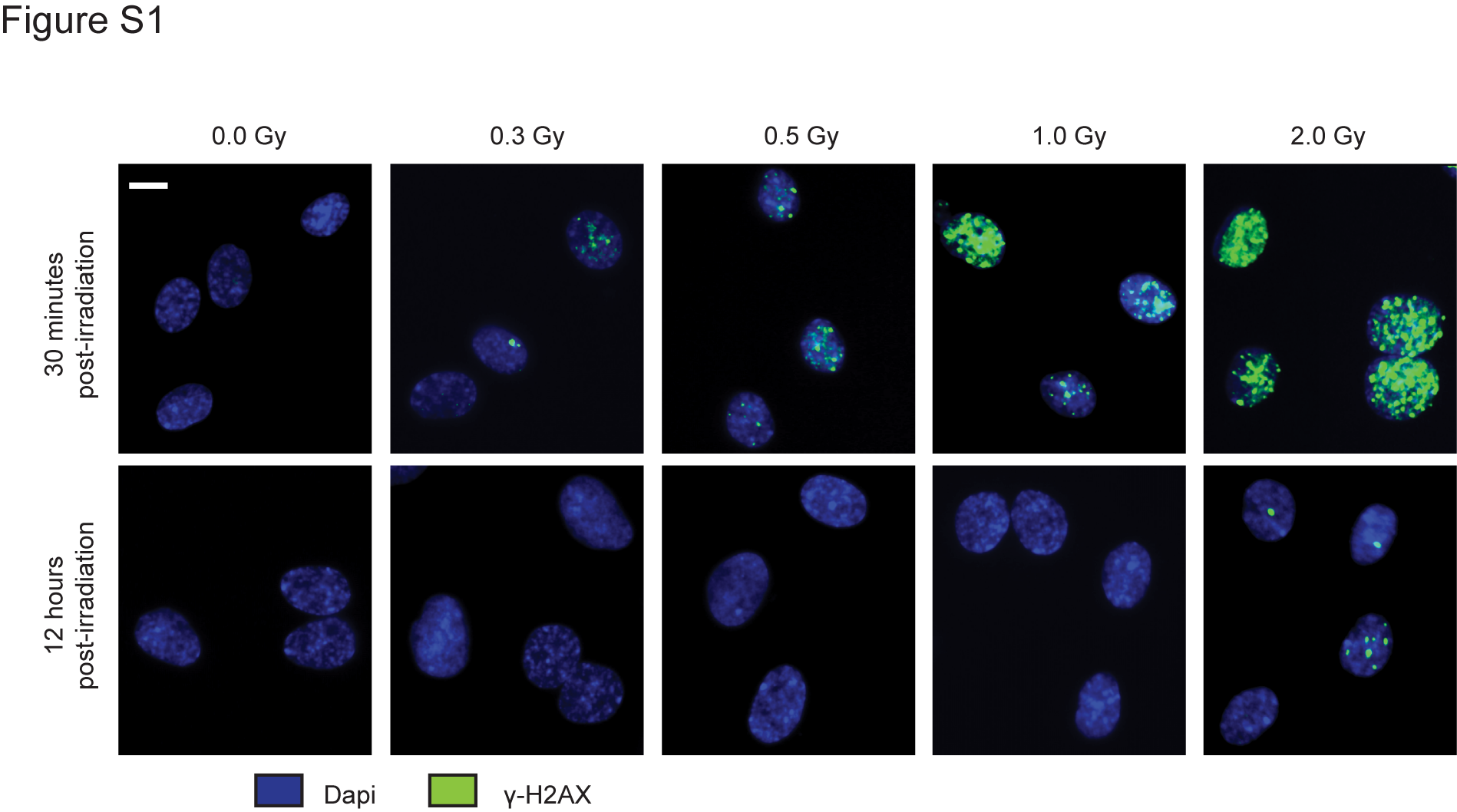

Supplement: Figure S1 — Low doses of IR induce phosphorylation of H2AX. Cells were exposed or not to 0.3, 0.5, 1.0 and 2.0 Gy and γ-H2AX foci, marking DNA damage, were visualized, by immunofluorescence microscopy, after 30 min and 12 h post-irradiation. γ-H2AX foci are shown in green and nuclei are stained with DAPI (in blue). Magnification, 400×. (1.07 MB TIF) [file pone.0011222.s001.tif]

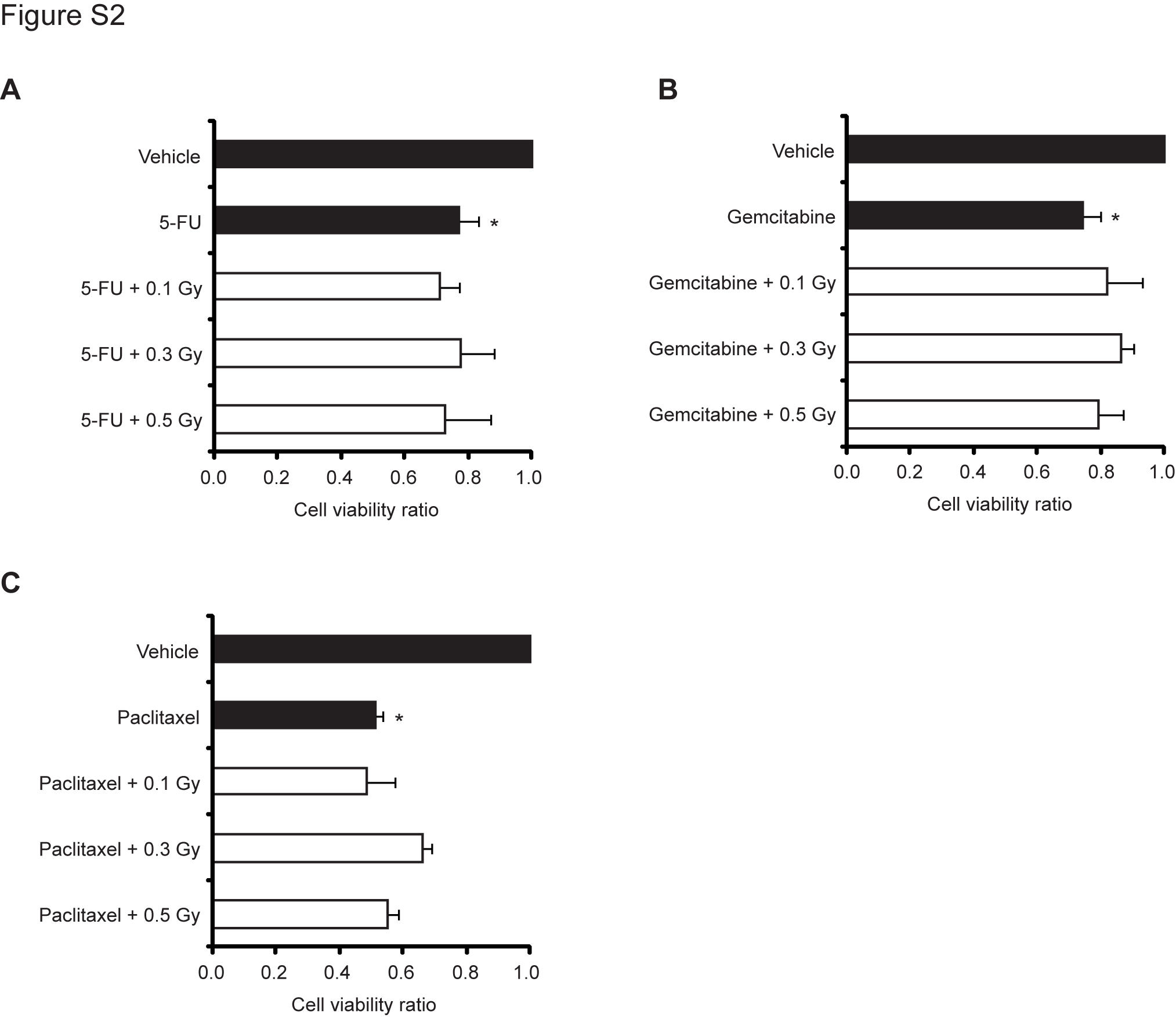

Supplement: Figure S2 — Low doses of IR do not protect the microvasculature from 5-FU-, gemcitabine- or paclitaxel-induced cell death. HMVEC-L were cultured for 12 h and treated or not with (A) 5-FU (5 µ g/ml); (B) gemcitabine (0.08 µ M); (C) paclitaxel (3 nM) and then exposed or not to 0.1, 0.3 or 0.5 Gy. Non-irradiated cells cultured with vehicle alone were used as a control. Cells were double stained with Annexin-V and propidium iodide at 48 h post-irradiation. The percentage of apoptotic cells was assessed by flow cytometry. Data (means ± s.d.) represent the ratio between the cell viability percentage of each experimental condition and the control condition and are derived from four independent experiments. (0.38 MB TIF) [file pone.0011222.s002.tif]

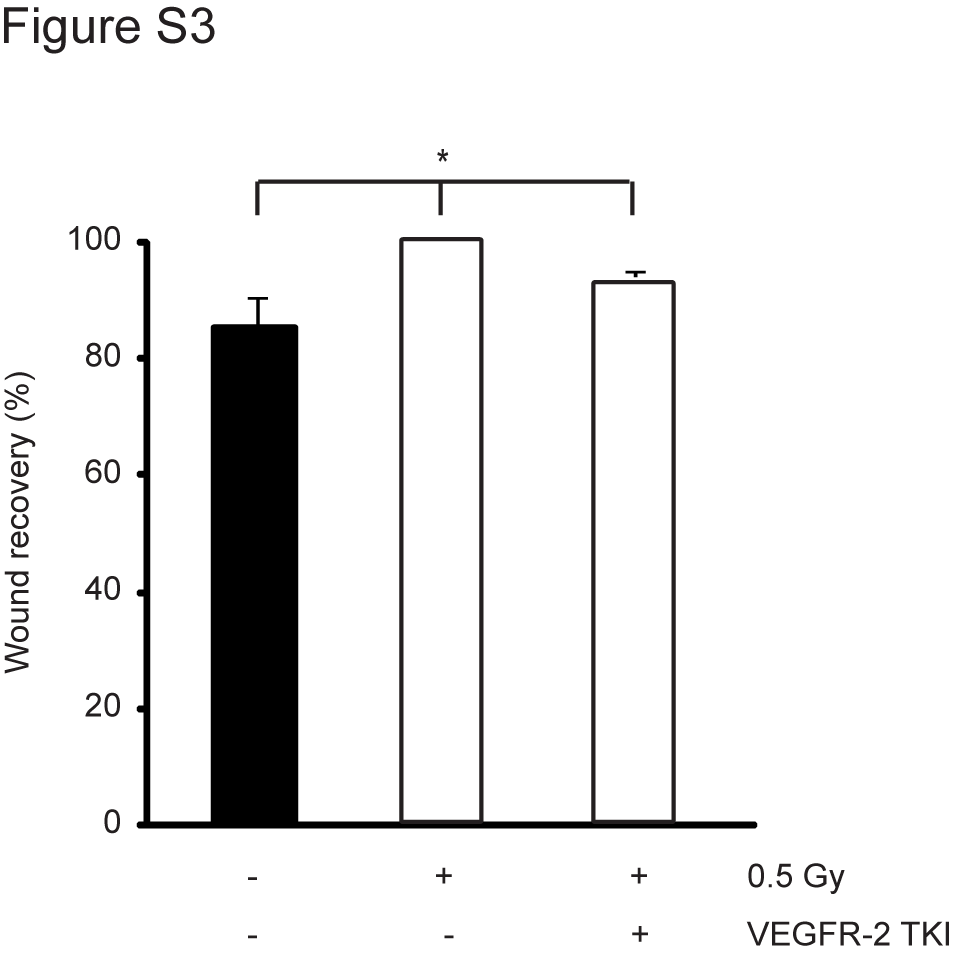

Supplement: Figure S3 — Low-dose IR promotes endothelial cell migration by activating VEGFR-2. Confluent monolayers of HMVEC-L were treated or not with VEGFR-2 tyrosine kinase inhibitor (TKI at 300 nM) for 2 h, subjected to in vitro wound healing and next exposed or not to 0.5 Gy. The quantification of the wound area (in mm2) was assessed 9h after wounding. Data (means ± s.d.) indicate the percentage of wound recovery in quadruplicate measurements and are representative of three independent experiments. *P<0.05. (0.13 MB TIF) [file pone.0011222.s003.tif]
